# Barriers and facilitators for shared decision making in older patients with multiple chronic conditions: a systematic review

**DOI:** 10.1186/s12877-021-02050-y

**Published:** 2021-02-06

**Authors:** Ruth E. Pel-Littel, Marjolein Snaterse, Nelly Marela Teppich, Bianca M. Buurman, Faridi S. van Etten-Jamaludin, Julia C. M. van Weert, Mirella M. Minkman, Wilma J. M. Scholte op Reimer

**Affiliations:** 1 Vilans, Centre of Expertise for Long-term Care, PO Box 8228, Utrecht, RE 3503 the Netherlands; 2 Department of Internal Medicine, Section of Geriatric Medicine, Amsterdam University Medical Center, University of Amsterdam, Amsterdam, the Netherlands; 3 ACHIEVE, Centre of Applied Research, Faculty of Health, Amsterdam University of Applied Sciences, Amsterdam, the Netherlands; 4 Medical Library, Amsterdam University Medical Centre, University of Amsterdam, Amsterdam, the Netherlands; 5 Amsterdam School of Communication Research/ASCoR, University of Amsterdam, Amsterdam, the Netherlands; 6 University of Tilburg/TIAS School for Business and Society, Tilburg, the Netherlands; 7 Department of Cardiology, Academic Medical Centre, University of Amsterdam, Amsterdam, the Netherlands

**Keywords:** Participation, Communication, Preferences, Personal experience, Informal caregivers

## Abstract

**Background:**

The aim of this study was to describe barriers and facilitators for shared decision making (SDM) as experienced by older patients with multiple chronic conditions (MCCs), informal caregivers and health professionals.

**Methods:**

A structured literature search was conducted with 5 databases. Two reviewers independently assessed studies for eligibility and performed a quality assessment. The results from the included studies were summarized using a predefined taxonomy.

**Results:**

Our search yielded 3838 articles. Twenty-eight studies, listing 149 perceived barriers and 67 perceived facilitators for SDM, were included. Due to poor health and cognitive and/or physical impairments, older patients with MCCs participate less in SDM. Poor interpersonal skills of health professionals are perceived as hampering SDM, as do organizational barriers, such as pressure for time and high turnover of patients. However, among older patients with MCCs, SDM could be facilitated when patients share information about personal values, priorities and preferences, as well as information about quality of life and functional status. Informal caregivers may facilitate SDM by assisting patients with decision support, although informal caregivers can also complicate the SDM process, for example, when they have different views on treatment or the patient’s capability to be involved. Coordination of care when multiple health professionals are involved is perceived as important.

**Conclusions:**

Although poor health is perceived as a barrier to participate in SDM, the personal experience of living with MCCs is considered valuable input in SDM. An explicit invitation to participate in SDM is important to older adults. Health professionals need a supporting organizational context and good communication skills to devise an individualized approach for patient care.

**Supplementary Information:**

The online version contains supplementary material available at 10.1186/s12877-021-02050-y.

## Background

There is much agreement that the prevalence of multiple chronic conditions (MCCs) has many negative consequences for older adults, such as functional impairment, a high treatment burden, a decline in health-related quality of life, increased use of health care and a higher risk of mortality [1–9]. Therefore, for many older adults with MCCs, maintaining (functional) independence, reducing symptom burden and acquiring emotional health and safety might be more important health outcomes than disease-specific outcomes [10]. The best treatment for the disease might not be the same as the best treatment for the patient as a whole. However, this requires another style of health care communication: instead of focusing on the treatment of each individual condition, the conversation should start with exploring an older adult’s priorities regarding preferred health outcomes, thus guiding the discussion of options and decisions about treatment or care. Since both the personal preferences of the older adult and the professional experience of the health professional are needed, this process is called ‘shared decision making’.

Shared decision making (SDM) facilitates the discussion between health professionals and older patients with multiple chronic conditions (MCCs) when decisions have to be made about the desired care and treatment. Elwyn (2017) describes SDM as “a process in which decisions are made in a collaborative way, where trustworthy information is provided in accessible formats about a set of options, typically in situations where the concerns, personal circumstances, and contexts of patients and their families play a major role in decisions [11]. The outcomes of SDM mainly report on cognitive-affective outcomes of SDM, such as knowledge and decisional conflict, and the evidence points towards positive effects of SDM in this perspective [12–14]. In particular the many studies about the use of patient decision aids provide evidence about better informed patients [12, 15]. There are fewer studies about behavioral outcomes such as compliance to treatment or adoption of health behaviors and about health outcomes such as quality of life [14]. Also the evidence in those studies directs less clearly to positive effects of SDM [16, 17].

SDM is not yet common practice; it is estimated that in only 10% of the situations in which health decisions have to be made, SDM is used [18]. Both health professionals and patients experience barriers in making shared decisions. Most reviews focus on SDM in a general population [19–21]. One review reveals barriers and facilitators of SDM in the daily life of people with dementia [22]. However, we expect that when facing decisions, older patients with MCCs and their informal caregivers may encounter additional barriers and facilitators, which should be identified to support the implementation of SDM [12, 23]. For example, characteristics such as anxiety, low health literacy and frailty are highly prevalent among older adults with MCCs and may influence the SDM process [24–28]. Anxiety is highly prevalent among older adults and associated with MCCs [24, 25]. Anxiety in SDM may leave the patient wanting to surrender decision making to the clinician [29]. Low HL is especially prevalent among older adults, with rates of low health literacy ranging from 30 to 68% [27, 30, 31]. The prevalence of low HL increases when there are MCCs [27]. Low HL among older adults is associated with poor shared decision making ability [27]. Older adults with MCCs who lack the ability to understand and communicate information may have trouble participating in parts of the SDM process, such as interpretation of test results and understanding the risks and benefits of procedures, leading to uncertainty and decisional conflict [27, 28]. It is estimated that approximately 20–30% of adults over 75 years are frail [32]. For adults who are frail, balancing benefits and harms of a treatment is important, since resilience capacity is often low. Furthermore, the presence of an informal caregiver, such as a family member or friend, at a medical consultation is common among older adults with MCCs. For example, in our observational study we found that in 63% of the geriatric consultations older adults were accompanied by informal caregivers [33]. Informal caregivers are often involved in discussing the patients health situation and participate in decision making [34]. Their role becomes more substantial when older patients are less able to participate in the consultation, for example in cases of cognitive decline [35–37]. Therefore, SDM with older adults with MCCs often has a triadic character, in which older patients with MCCs, their informal caregivers and health professionals participate.

**Fig. 1:**
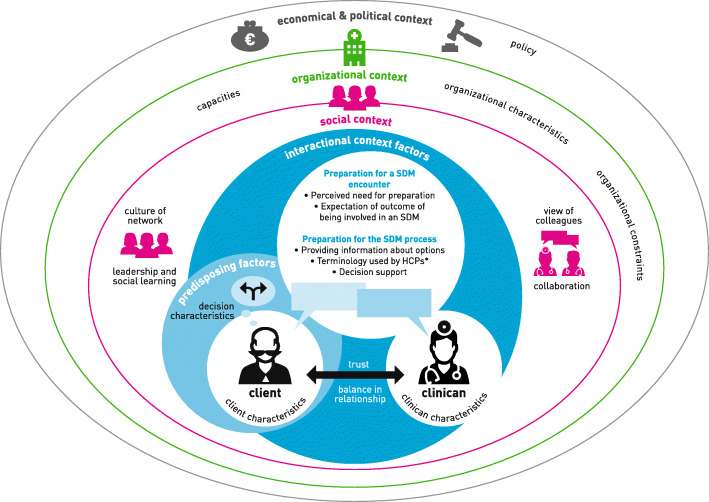
Adapted taxonomy for barriers of and facilitators to shared decision making

In a previous review about patient-reported barriers and facilitators to SDM a taxonomy of barriers and facilitators to SDM was developed [19]. In this taxonomy (see Supplementary Table S2) barriers and facilitators were coded into the following categories: predisposing factors (patient and decision characteristics), interactional context factors (social factors regarding the relation between patients and health professionals), preparation for the SDM encounter (perceived need for preparation by patients and expectations about involvement) and preparation for the SDM process (providing information about options, decision support and terminology used). To gain more insight into the implementation of SDM, we enriched this taxonomy with organizational factors (health care organizations), social factors (health care settings, interdisciplinary team) and policy factors (health care system, health government) as reported by Grol et al. [38]. To explain the taxonomy, we developed Fig. 1, which visualizes the adapted taxonomy. From all three perspectives (patient, informal caregiver and health care professional), barriers and facilitators could be reported for all types of factors.

The aim of this study is to conduct a systematic review to identify barriers and facilitators that older patients with MCCs, their informal caregivers and health professionals experience in SDM.

## Methods

This systematic review followed the Preferred Reporting Items for Systematic Reviews and Meta-analysis (PRISMA) statement [39].

### Search strategy

We searched five electronic databases (Medline, EMBASE, PsycINFO, Cinahl, and Cochrane Central Register of Controlled Trials (Central)). Because the concept of SDM was not widely spread until the early 1990s, our search covers the period from 1980 to January 1, 2019. Based on a list of 20 key articles in the field of barriers and facilitators to SDM, the clinical librarian developed a search strategy (Supplementary Table S1). We used both keywords and MeSH terms for ‘shared decision making’, ‘older patients’, ‘multiple chronic conditions’, ‘barriers’ and ‘facilitators’.

### Eligibility criteria

A study was eligible for inclusion if 1) it was an original collection of data, 2) the design targeted older people (mean age > 65 years) with MCCs (> 2 chronic conditions), 3) the results reported perceived barriers and/or facilitators for SDM, and 4) the study focused on either the perspective of patients and/or that of informal caregivers, health professionals or both. Health professionals were defined as medical staff, nurses and other professions allied to medicine, e.g. clinical psychologists, dieticians, physiotherapists. Conference/poster abstracts and articles that could not be retrieved were excluded.

### Study selection

First, titles and abstracts, and second, full-text versions of potentially relevant articles were screened independently by two authors (RP, NT) on the basis of the eligibility criteria. Disagreements were resolved through discussion with a third reviewer (MS).

### Data extraction and quality assessment

Information about the characteristics of the studies (type, setting) and perceived barriers and facilitators to SDM were extracted independently by two reviewers (RP, NT) using a data extraction sheet. Data synthesis was achieved using deductive content analysis. The reviewers identified each unit of text (a paragraph or sentence depicting one idea) relevant to the main outcomes (barriers or facilitators to SDM). Each unit of text was subsequently coded according to the taxonomy of barriers and facilitators to SDM. Two researchers (RP, NT) independently coded all retrieved units of text, and any discrepancies between the codes were resolved through discussions.

Similar to other reviews about facilitators and barriers for SDM, the quality of the included studies was assessed using the Standard Quality Assessment Criteria for Evaluating Primary Research Papers from a Variety of Fields (SQAC) [19, 21, 40]. The quality scores of the SQAC were used to define a minimum threshold for the inclusion of studies. Following the SQAC manual, the cut-point for exclusion was set at <.55 (range 0–1). All studies were independently assessed by two researchers (RP, NT), and disagreements were resolved through discussion with a third reviewer (MS).

## Results

### Study selection

**Fig. 2:**
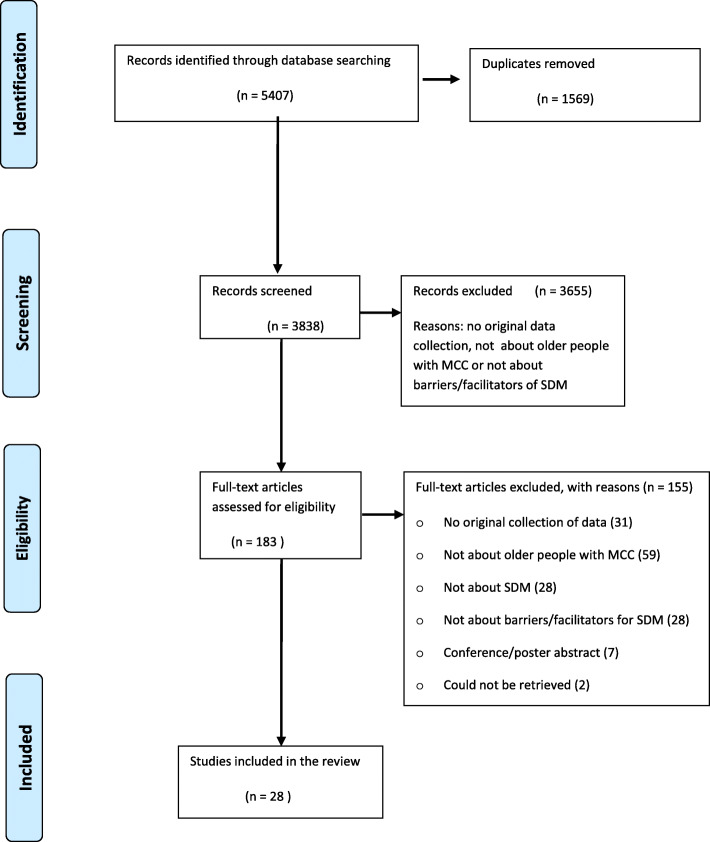
PRISMA flow diagram of literature review process for studies on barriers of and facilitators to shared decision making in older patients with multiple chronic conditions

The database searches generated 3838 unique abstracts. After screening titles and abstracts, 183 full texts were reviewed, of which 28 studies met the inclusion criteria (Fig. 2).

### Study characteristics

The included studies (Table 1) comprised 2990 older adults, 337 informal caregivers (IC) and 527 health professionals (HCPs). The studies originated from the U.S.A. (*n* = 13), Canada (*n* = 4), Sweden (*n =* 4), Norway (*n =* 2), the Netherlands (*n =* 2), Australia (*n =* 1), Germany (*n =* 1) and the U.K. (*n =* 1). Nineteen studies used a qualitative design [41, 42, 44, 46, 47, 49–51, 54, 55, 57, 59–63, 65, 66, 68], five studies used a quantitative design [43, 45, 48, 58, 64] and four studies used a mixed-method design [52, 53, 56, 67]. Neither the quantitative nor the mixed-methods studies carried statistical analysis out regarding the barriers and facilitators, they all reported in a qualitative way on the perceived barriers and facilitators. The 28 studies reported perceived barriers and facilitators from different stakeholder perspectives: nine studies (32%) reported on the patient perspective [41–49], three studies (11%) focused on the informal caregiver perspective [57–59], eight studies (29%) reported on the health professional perspective, and 7 studies (25%) reported more than one perspective [50–56]. Decisions were about medical treatment [43, 45, 47, 48, 50, 60, 61, 64, 67], medication [44, 65], goals of care [50, 53, 62, 63, 66], daily life and lifestyle [41, 50, 58, 64], hospital admission or discharge [46, 57, 68] and ethical or end-of-life dilemmas [42, 49].

**Table 1:** Characteristics of the included studies

First author	Setting	Country	Design study	Reporting focus^**a**^: Barriers (B) and/or Facilitators (F)	Perspective^**b**^	Participants (n)	% Female	Mean age of patients (years) (if not available: age range)
Funk [41], 2004	LTC facilities	Canada	Qualitative	B&F	Patients	100 patients	82	85
Gauthier [42], 2005	hospital	U.S.A.	Qualitative	B&F	Patients	13 patients	62	72
Naik [43], 2011	primary care	U.S.A.	Quantitative	B	Patients	100 patients	100	71
Belcher [44], 2006	primary care	U.S.A.	Qualitative	B&F	Patients	51 patients,	63	77
Chi [45], 2017	community care	U.S.A.	Quantitative	B	Patients	2017 patients	57	range 65 > 85
Dyrstad [46], 2015	hospital	Norway	Qualitative	B&F	Patients	41 patients	46	86
Ekdahl [47], 2010	hospital	Sweden	Qualitative	B&F	Patients	15 patients	67	84
Ekdahl [48], 2011	hospital	Sweden	Quantitative	B	Patients	156 patients	49	83.1
Erickson [49], 1989	community care	U.S.A.	Qualitative (case study)	B	Patients	1 patient	100	75
Petrillo [50], 2018	hospital	U.S.A.	Qualitative	F	Patients and informal caregivers	38 patients 31 informal caregivers	48 (total group)	78
Riffin [51], 2018	primary care	U.S.A.	Qualitative	B	Patients and informal caregivers	20 patients 20 informal caregivers	61 65	82
Kiselev [52], 2017	hospital & community care	Germany	Mixed methods	B&F	Patients and health professionals	283 patients 14 professionals (clinicians, nurses, therapists, psychologist, social worker)	66 unk^c^.	74.4
Rose [53], 2018	rehabilitation	U.K.	Mixed methods	B	Patients and health professionals	40 patients 24 professionals (rehabilitation staff)	23 unk.	83
Ferris [54], 2018	health care users and providers	U.S.A.	Qualitative	B&F	Patients, informal caregivers and health professionals	6 patients or informal caregivers 30 clinicians, health systems, and payers	unk.	unk.
Doekhie [55]	primary care	The Netherlands	Qualitative	B	Patients, informal caregivers and health professionals	19 patients 10 informal caregivers 38 professionals (clinicians, nurses, paramedics)	79 40 unk.	81.6
Puts [56], 2017	hospital	Canada	Mixed methods	B	Patients, informal caregivers and health professionals	29 patients 24 informal caregivers 28 professionals (oncologists and family physicians)	24 resp. 36	patients divided in 2 age groups: 63–79 & > 80;
Bragstad [57], 2014	hospital	U.S.A.	Qualitative	B	Informal caregivers	19 informal caregivers	68	n.a.^d^
Menne [58], 2008	community care	U.S.A.	Quantitative	B&F	Informal caregivers	215 informal caregivers	50	n.a.
Peacock [59], 2017	community care	Canada	Qualitative	B	Informal caregivers	18 informal caregivers	44	n.a.
Ekdahl [60],2012	hospital	Sweden	Qualitative	B&F	Health professionals	29 physicians	34	n.a.
Fried [61], 2011	primary care	U.S.A.	Qualitative	B	Health professionals	40 physicians		n.a.
Blaum [62], 2018	primary care & hospital	U.S.A.	Qualitative	B	Health professionals	9 general practitioners, 5 cardiologists		n.a.
Gopalraj [63], 2012	hospital	U.S.A.	Qualitative (case study)	B&F	Health professionals	1 patient	100	94
Milte [64], 2015	geropsychiatry inpatient unit	Australia	Quantitative	B	Health professionals	2 geriatricians	59	n.a.
Schuling [65], 2012	hospital	The Netherlands	Qualitative	B	Health professionals	13 physicians	15	n.a.
Molinari [66], 2016	geropsychiatry inpatient unit	U.S.A.	Qualitative (case study)	F	Health professionals	1 patient	0	‘late 60s’
Légaré [67], 2013	primary care	Canada	Mixed methods	B&F	Health professionals	Participants: a) 276 home care providers b) 7 members health care team c) 8 managers	Participants: a) 82 b)100 c) 50	n.a.
Lindhardt [68], 2008	hospital	Sweden	Qualitative	B&F	Health professionals	8 nurses	100	n.a.

^a^‘Reporting focus’ refers to whether the study reports about perceived barriers to and/or facilitators of SDM

^b^‘Perspective’ refers to either the perspective of patients or informal caregivers or health professional from which the perceived barriers or facilitators are reported. Some studies describe perceived barriers or facilitators from more than one perspective

^c^*unk* unknown

^d^*n.a* not applicable

Ten studies were based in a hospital setting [46–48, 50, 56, 57, 60, 63, 65, 68], six in a primary care setting [43, 44, 51, 55, 61, 67], four in a community care setting [45, 49, 58, 59, 69], one in a long-term care setting [41], one in a hospice [42], one in a post-acute residential care setting [64], one in a rehabilitation setting [53], and one in a geropsychiatric inpatient unit [66]. Three studies were based in a combined setting, e.g., hospital and primary care [52, 54, 62]. The study patients’ age for each study is depicted in Table 1. In all studies, patients had > 2 diagnoses, although in one study, a subgroup of patients had < 2 diagnoses [45].

### Quality assessment

Supplementary Table S3 shows the quality assessment scores of the included studies. All qualitative studies scored > .55 and thus met the quality standard. However, three qualitative studies [49, 63, 66] were case studies and could not be assessed within the SQAC format. All the quantitative studies scored > .77. The mixed-method studies had a summarized score > 0.80 (see Supplementary Table S3).

### Barriers and facilitators of SDM for older patients with MCCs

A comprehensive overview of all barriers and facilitators found is presented in Table 2. From the twenty-eight included studies, we found 149 perceived barriers and 67 perceived facilitators for SDM in older patients with MCCs. Most barriers were found in the following categories: predisposing factors (*n* = 51, 34%), interactional context factors (*n* = 32, 21%) and organizational context factors (*n* = 22, 15%). Most facilitators were found in the following categories: interactional context factors (*n* = 23, 34%) and preparation for the SDM process (*n* = 19, 28%). In Table 2 is also depicted how many studies reported about a specific barrier or facilitator, to provide insight into how often a factor was reported. In the next section the most frequently mentioned barriers and facilitators are described and explained from which perspective the barriers and facilitators were reported: patient perspective (PP), informal caregiver perspective (IP) or health professional perspective (HP).

**Table 2:** Barriers and facilitators for SDM in older patients with MCCs

Factor		Barriers (number of studies in which this factor was identified as a barrier)	Facilitators (number of studies in which this factor was identified as a facilitator)
Predisposing factors	Patient characteristics	Being in poor health: 13 Cognitive/physical impairments: 13 Lower level of education: 5 Age: 4 Poor articulation: 4 Difference in personal characteristics: None Health condition - stigma/discrimination: 2 Ethnicity: 1 Long term patient: 1	Prior exposure to illness/decision making point: 4 Personal values: 1 Being in good health: 1 Long term patient: None^a^
	Decision characteristics	Disease-based decision models (guidelines): 3 Burden of treatment regimen: 2 Shock of receiving diagnosis: 2 Minor decision: 1 Timing along the illness trajectory: None Major decision: None Embarrassing or sensitive topics: None	When decisions are allowed that are inconsistent with guidelines: 1 Major decision: 1 Timing along the illness trajectory: None Minor decision: None Time to come to terms with diagnosis: None
Interactional context factors	Power (im) balance in the patient-clinician relationship	Presumptions about the patient role Not having explicit ‘permission’ to participate in SDM: 6 Expectation of the clinician making the decisions: 2 Desire to act as a ‘good’ patient (driven by fear of consequences): 1 Belief that clinicians do not want patients involved: 1 Perceived acceptability of asking the clinician questions: 1 Clinicians reinforces passivity by rewarding the behaviour: None Patients undervalue their expertise relative to clinicians ‘Doctor knows best’ and patients have ‘inferior’ knowledge: 3 Patients are not capable of understanding medical/technical information: 2	Presumptions about the patient role Having explicit ‘permission’ to participate in SDM: 4 Perceived acceptability of asking the clinician questions: None Patients undervalue their expertise relative to clinicians Recognizing there are two experts in the medical encounter: 5
	Interpersonal characteristics of the clinician	Clinicians with poor interpersonal skills: 5 Authoritarian HCPs: 4 Clinician does not listen to patients concerns: 2 Perceptions that clinicians are already doing SDM: 1 Lack of individualized approach and not asked about preferences: 1 Clinician does not address patient directly: 1 Poor relationship with clinician: None	Individualized approach where clinician seeks patient’s preferences: 4 Clinicians with positive interpersonal skills: 2 Equal relationship: 1 Clinician listens to patients concerns: 1 Good relationship with clinician: None
	Trust	Trust in clinician: None Lack of trust in clinician: 2	Trust in clinician: 6 Lack of trust in clinician: None
Preparation for an SDM encounter	Perceived need for preparation	Patient does not *want* or *need* to participate in SDM: 4 Patient is not entitled to a choice: 1 Patient is not explicitly offered a choice or it is presented in a biased way: 1 ‘Doing nothing’ is not an option: None Not knowing what to expect from the SDM consultation: None	Accepting responsibility to be involved in decision-making: 5 Setting an agenda: 1
	Expectation of SDM outcomes	Patient focus on treatment burden versus clinicians concerns about morbidity and mortality: 2 Not wanting responsibility for wrong decision: 1 Fear of accepting reality of diagnosis: None	Recognizing equipoise and uncertainty: 1
Preparation for the SDM process	Providing information about options	Insufficient information about condition, options and outcomes: 3 Clinician does not explain the options and outcomes: 2 Clinician in repair-reflex mode (solutions without listening to patient’s preferences): 1 No flexibility of clinicians when patients want something different: 1	Sufficient information about condition, options and outcomes: 5 Clinician explains the options and outcomes: 2 Clinician knows patient’s and informal caregivers’ priorities, goals and preferences: 1
	Terminology used by HCPs	Clinician uses medical terminology: 1	Clinician uses simple terminology: 1
	Decision support	Decision support from informal caregivers: 4 Lack of written decision support: 1 Purpose of decision support tool is unclear: None	Decision support from others (e.g., family, other professionals): 15 Written decision support: None
Social context	View of colleagues	Disagreement between colleagues: 3 Degree of contact between colleagues: 1 Hierarchical structure of professionals: 1	
	Culture of network	Social norms and values: None	Social norms and values: None
	Collaboration	Degree of cooperation and response between colleagues: 10	Degree of cooperation and response between colleagues: 6
	Leadership and social learning	Lack of support from management (incentive, feedback, role models): 3	Support from management (incentive, feedback, role models): None
Organizational context	Organizational characteristics	Complexity of the organization: 4	Complexity of the organization: 1
	Capacities	No arrangements for continuous learning: 1	Continuous learning opportunities: None
	Organizational constraints	Lack of resources (time): 11 Lack of support services: 2 Lack of resources (staff): 4	Lack of resources (time): 3 Lack of support services: None Lack of resources (staff): None
Economic and political context	Policy	Unattractiveness of innovation by means of financial arrangements: 2	Attractiveness of innovation by means of financial arrangements: 1
Other		6	None

^a^‘None’ refers to the fact that no barrier or facilitator was found for this factor

### Predisposing factors

#### Perceived barriers

When one is very ill, this affects one’s ability to understand information (HP/PP) [46–48] and to participate in decision making (PP) [42, 48]. Patients suffering from MCCs are less likely to participate in SDM and worry about the burden of a treatment regime (PP/IP/HP) [45, 54, 59, 61]. Cognitive and physical impairments were considered barriers for SDM by patients, informal caregivers and health professionals. Cognitive impairment leads to difficulties in understanding information (PP/IP/HP) [47, 48, 58, 60, 63], not being able to express preferences (HP) [60], and not wanting (HP) [64] or not being able (PP) [47] to partake in decision making. Physical impairments can influence compliance (HP) [61], whereas severe illness (PP) [42] can keep older patients with MCCs from being able to partake in decision making. Health professionals often struggle with the uncertainties of applying disease-specific guidelines to older patients with MCCs (PP/IP/HP) [54, 61, 65]. For information about the exact numbers of articles reporting barriers, we refer to Table 2.

#### Perceived facilitators

Previous experience in dealing with conditions and decision making acts as a facilitator to SDM (PP) [41, 44, 46]. Additionally, having personal values, such as religion, views on survival and suffering, and self-sufficiency facilitates the SDM process (PP/IP) [50]. For information about the exact numbers of articles reporting facilitators, we refer to Table 2.

### Interactional context factors

#### Perceived barriers

Both patients and health professionals reported poor communication techniques, poor language choice and lack of empathy as barriers for shared decision making (PP/HP) [44, 47, 63]. Older patients had little confidence that they could have a meaningful contribution to the shared decision-making process, or they felt that a lack of (medical) knowledge made them unable to participate (PP) [41, 44]. On the other hand, some patients reported feeling that health professionals undervalue the expertise of patients (P) [55]. Informal caregivers expressed dependency; they felt they were at the mercy of the individual health care personnel and that, in the end, the final decisions were made by the health professionals (IP/PP) [46, 57]. Health professionals acknowledged that most of the time patients and informal caregivers are not seen as part of the health care team (IP/PP/HP) [46, 47, 55, 57].

#### Perceived facilitators

Health professionals report that clinicians who assessed a patient’s ability to understand information and to describe his or her symptoms, thoughts and feelings, particularly for patients with cognitive decline, facilitated SDM (HP) [60, 63]. Patients reported that they did feel invited to partake in shared decision making when the doctor stimulated them to describe symptoms and ask questions and inquired what the patient’s main worries were (PP/HP) [44, 46, 64]. Additionally, holding the belief that they are the ones with the most knowledge about their own body and particular conditions facilitated active involvement of older patients (PP) [44].

### Preparation for an SDM encounter

#### Perceived barriers

Not all patients want or need to participate in SDM (HP/PP) [41, 47, 60]. A segment of the older patients preferred a more passive role in SDM (HP/PP) [41, 47, 60]. Health professionals, however, viewed a lack of participation as a barrier to SDM because they feel uncomfortable when they have to guess the patient’s preferences (HP/PP) [47, 60]. Different views may complicate SDM, e.g., patients focus on treatment burden versus clinicians concerns about morbidity and mortality (HP) [61, 62].

#### Perceived facilitators

Patients feel they have an own responsibility in asking questions, learning about their disease and medications and inquiring on investigations and medical considerations (PP) [44, 47]. Also, patients suggested that motivation, self-confidence, preparing themselves and family support could enable them to participate in SDM (PP/HP) [53].

### Preparation for the SDM process

#### Perceived barriers

When health professionals did not adapt information to the personal needs and capacities of patients or used medical terminology, their behaviours were experienced as a barrier to SDM (PP) [46, 48]. Decision support from informal caregivers can also be a burden to SDM. First, informal caregivers sometimes feel forced responsibility in decision making (IP) [59]. Second, there can be different views between informal caregivers and health professionals, e.g., informal caregivers being overprotective or acting against professional advice (PP/IP/HP) [55]. Third, there can be conflicting views between informal caregivers and patients about treatment or care but also about the ability of the patient to communicate adequately with the health professional (PP/IP/HP) [51, 55, 59]. This might occur particularly in cases of cognitive decline. Finally, SDM can be complicated when there is not one but more than one informal caregiver involved, sometimes each with a different opinion (PP/IP/HP) [55].

#### Perceived facilitators

When patients had cognitive decline or were too ill, informal caregivers supported the decision-making process by providing information, asking questions and assisting in receiving and understanding information (PP/HP) [42, 44, 46, 60, 64, 68]. In addition, supportive informal caregivers ensured that patient preferences were recognized [51]. Furthermore, personal experiences of family and friends are important in balancing options (PP/IP/HP) [50, 56]. Tailored information about conditions, options and outcomes was experienced as a facilitator to SDM (PP/HP) [44, 64]. Health professionals state that information about the patients’ quality of life and functional status and knowledge about patient and informal caregivers’ priorities, goals and preferences facilitates SDM (PP/IP/HP) [54].

### Social context

#### Perceived barriers

Care for older patients with MCCs often involves many different types of health professionals often working in different health care settings. Poor or inefficient communication between them, leading to difficulty in prioritization and no one having the overview of a patient’s case, was experienced as a barrier to SDM and to the need for integrated care in general (PP/IP/HP) [47, 55, 60, 61, 63]. Differences in vision, for example, medical focus versus focus on wellbeing, or conflicting ideas about patient involvement hamper SDM (PP/IP/HP) [53, 55, 67, 68].

#### Perceived facilitators

Conversely, good cooperation, communication and the use of the same vocabulary among the interdisciplinary team members facilitated SDM (HP) [61, 63, 67].

### Organizational context

#### Perceived barriers

Studies reporting organizational barriers were mostly situated in hospital settings (*n* = 8) or in primary care settings (*n* = 2). A high turnover in staff makes it difficult to get to know older patients and results in a lack of continuity of care and situations in which it is unclear who is responsible for the patient (HP) [60, 67, 68]. Lack of a good electronic patient record results in the patient having to frequently repeat explanations (PP/IP/HP) [54]. One study reported that patients sometimes felt urged to make room for new patients and that so-called ‘shared decisions’ about discharge were actually made solely by the professionals (PP) [46]. Discussing the personal preferences of older patients requires a relationship between the patient and clinician, and time is necessary to establish such a relationship. One study reported that when patients felt that the staff was stressed, they experienced less ability to participate in decision making (PP) [47].

#### Perceived facilitators

When the workflow is genuinely organized around the patient, this facilitates SDM (PP/IP/HP) [54].

### Economic and political context

#### Perceived barriers

When there is a system of payment for productivity, indicating that payment is only indicated when a medical treatment or intervention is chosen, this hampers the SDM process since choosing a treatment is then rewarded above watchful waiting (PP/IP/HP) [54]. Additionally, formal re-imbursement rules limit choices for patients (PP/HP) [52].

#### Perceived facilitators

On the other hand, a value-based payment system facilitates SDM, because the payment is then related to the outcomes relevant to the patient, which can range from comprehensive medical treatment to watchful waiting (PP/IP/HP) [54].

### Other perceived barriers

Six barriers did not fit into the above framework. Patients mentioned having intense emotions, such as anger and frustration (PP) [42], and having a constantly changing medical condition, leading to difficulties in keeping up with information (PP) [44], as barriers for SDM.

### Differences in perspectives between patients, informal caregivers and health professionals

**Fig. 3:**
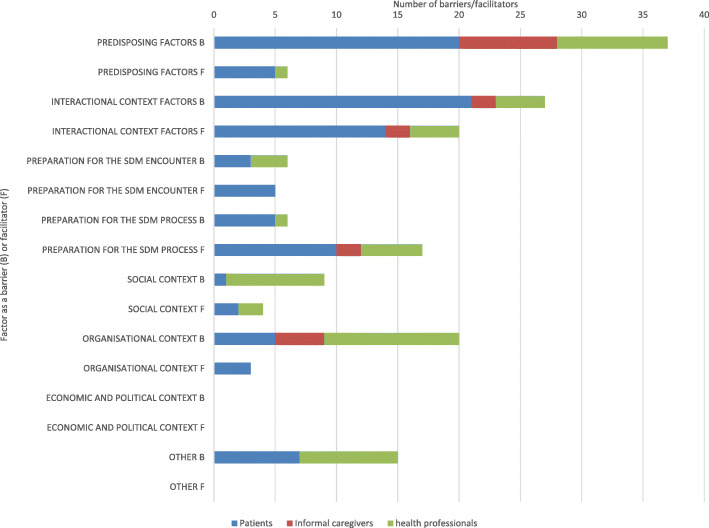
Barriers of and facilitators to shared decision making as experienced by patients, informal caregivers and health professionals

Fig. 3 demonstrates how the main barriers and facilitators in this review were experienced from three different perspectives: patients, informal caregivers and health professionals. Almost all barriers and facilitators were reported from more than one perspective. For example, poor health is experienced as a barrier to SDM by patients, informal caregivers and health professionals.

The main barriers from a patient’s perspective were found in predisposing factors (*n* = 24, 20%) and interactional context factors (*n* = 23, 19%). Informal caregivers experienced most barriers in predisposing factors (*n* = 10, 50%), while health professionals reported mainly barriers in predisposing factors (*n* = 17, 22%) and in the organizational context (*n* = 12, 15%).

## Discussion

We identified 28 studies papers reporting on perceived barriers and facilitators about SDM in older patients with MCCs. The main barriers for SDM as experienced by older patients with MCCs are ascribed to personal patient characteristics, such as poor health and/or cognitive or physical impairments. MCCs might complicate SDM in several ways: patients with MCCs experience less participation in SDM and health professionals experience difficulties with single diseased-based guidelines. Furthermore, patients and health professionals experience that differences in views on treatment burden versus morbidity and mortality complicates SDM. Additionally, with MCCs, there are often more health professionals involved, which may lead to conflicting views on treatment priorities, patient and caregiver involvement and no one coordinating and integrating patient care. Health professionals perceive poor interpersonal skills as a barrier to SDM as well as organizational barriers, such as pressure for time and high turnover of patients. Financial incentives, such as payments for productivity, are experienced as counterproductive to SDM, when ‘doing nothing’ is also an important option to discuss. However, older patients with MCCs perceive that SDM is facilitated when patients share information about personal values, priorities and preferences, and information about quality of life and functional status. Decision support by informal caregivers is perceived as a facilitator to SDM, although informal caregivers can also complicate the SDM process, for example, when an informal caregiver has different views on treatment or about the patient’s capability to be involved. The main experienced facilitator for SDM is an individualized approach in which health professionals probe and prioritise patient preferences. Coordination of care when multiple health professionals are involved seems important.

Most of the main perceived barriers and facilitators for SDM were reported from more than one perspective. There was great overlap between patients and health professionals in what they perceived as helping or hindering SDM. Commonly experienced, frequently reported barriers included patient characteristics (poor health, cognitive impairments), poor communication techniques by health professionals and organizational constraints (e.g., time pressure). Commonly experienced facilitators were acknowledgement of the complex conditions of patients by health professionals and the effect of this factor on participation in SDM.

These findings should be considered in relation to other reviews about the implementation of SDM. We found that poor health and cognitive impairment in older patients are perceived barriers to participation in SDM, whereas other reviews do not reveal these factors as important barriers. This observation suggests that the presence of MCCs in old age requires more effort from health professionals to engage patients in SDM. The review of Joseph-Williams (2014) emphasizes the importance of knowledge to patients when participating in SDM; patients often feel insecure about their own medical knowledge and undervalue their knowledge about their personal situation and experiences [19]. The current review confirms that patients often underestimate their own expertise [70] but, in contrast, reveals that due to their MCCs, they feel more experienced in using health care facility systems than those with single health conditions [41, 46] and perceive that because of the chronic aspect of their conditions, they have greater knowledge about their particular condition and preferences [44]. Feeling no permission to participate in SDM is also mentioned in the review by Joseph-Williams and is consistent with our findings. A review of the key components of SDM models found that only about one third of SDM models includes ‘discussing the preferred roles of patients’ and ‘communicating that the patients’ opinion is important’ [71]. Elwyn et al. (2017) transformed the first step of their SDM model ‘Choice talk’ to ‘Team talk’, emphasizing the importance of explaining the intention to collaborate and support deliberation [11]. During the development of the ‘Dynamic model for SDM in frail older patients’ [72], patients stressed the importance of being engaged in the dialogue [72].

Consistent with previous research, we found that professionals perceive a lack of agreement on the SDM process or SDM aids [20, 73]. In our study, this is ascribed to the involvement of multiple professionals in the case of patients with MCCs. Although the aspect of time is also described in existing reviews about the implementation of SDM [19–21, 73], the findings in this review stress that health professionals experience that more time is needed to establish a relationship with older patients.

This review also addressed the informal caregivers’ perspective on SDM. Echoing previous research, we found that decision support from informal caregivers is experienced as a considerable facilitator to SDM [35–37], however, there are several ways in which decision support from informal caregivers may also pose a burden on SDM [51, 55, 59].

Our findings should also be interpreted in the broader context of SDM developments. This study highlights that for older adults with MCCs an individualized approach is needed, taking into account the personal experience of patients that live with chronic conditions facilitate SDM. These personal experiences may direct the discussion about patients’ personal preferred health outcomes. This in line with the ‘Action Steps for decision making for older adults with MCCss, according to the MCCs guiding principles, that emphasizes to start with identifying and communicating patients’ preferences and priorities [74]. Although older adults vary in whether they want and are able to participate in SDM, considering preferences is relevant for all patients [41, 47, 60, 74]. Tinetti (2019) found that working according to patients’ priorities led to less treatment burden and less unwanted healthcare [75]. They also reported that initial fear among physicians that patients would formulate unrealistic goals was unjustified; if patient were guided through the SDM process, they formulated personal and realistic goals. This was confirmed by the study of Feder (2019) who also found that discussing personal goals led to a better relationship with physicians [76].

We conducted a broad and systematic search; however, although we searched for studies about SDM in other health disciplines, most studies targeted clinicians. Furthermore, using an existing taxonomy has advantages and disadvantages. This taxonomy used in this study to structure barriers and facilitators was developed and used in previous reviews, thus making a comparison of the results possible. However, we found additional barriers and facilitators; those barriers were directly related to the characteristic features of SDM for older patients with MCCs, which was not a target population during the original development of the taxonomy. Barriers that were added to the taxonomy were ‘Disease-based decision models (guidelines)’, ‘Burden of treatment regimen’, ‘Patient focus on treatment burden versus clinicians concerns about morbidity and mortality’ and ‘Decision support from informal caregivers’. As facilitators were added: When decisions are allowed that are inconsistent with guidelines’ and ‘setting an agenda’.

## Conclusions

Although poor health is experienced as a barrier to participate in SDM, the personal experience of living with MCCs is perceived as valuable to SDM. Patients feel that an explicit invitation to participate in SDM is important. Informal caregivers would like to be respected as full partners in the SDM process; however, more research on their perspective is required. Health professionals expressed they need a supporting organizational context and good communication skills to work out an individualized approach for care. Finally, health professionals consider a value-based payment system as a facilitator to SDM unlike a payment-for-productivity system.

## Supplementary Information

**Additional file 1: Supplementary Table S1.** Medline via Ovid search strategy

**Additional file 2: Supplementary Table S2.** Taxonomy of barriers and facilitators

**Additional file 3: Supplementary Table S3.** Quality assessment of included studies

## Data Availability

The dataset(s) supporting the conclusions of this article are included within the article (and its additional files).

## Abbreviations

SDM: Shared decision making
MCCs: Multiple chronic conditions
PRISMA: Preferred reporting items for systematic reviews and meta-analysis
SQAC: Standard quality assessment criteria for evaluating primary research papers from a variety of fields
