# Supplementary material for: Barriers and facilitators for shared decision making in older patients with multiple chronic conditions: a systematic review
Source: BMC Geriatr. 2021 Feb 6;21:112. doi: 10.1186/s12877-021-02050-y (PMC7866443; doi:10.1186/s12877-021-02050-y)
Supplement: Supplementary file 1 — Additional file 1: Supplementary Table S1. Medline via Ovid search strategy [file 12877_2021_2050_MOESM1_ESM.docx]

# Supplementary Table S1: Medline via Ovid Search strategy search strategy

MEDLINE (Ovid)

Database(s): Ovid MEDLINE(R) In-Process & Other Non-Indexed Citations and Ovid MEDLINE(R) 1980 to 1-1-2019 
Search Strategy:

| # | Searches |
| --- | --- |
| 1 | decision making/ or Patient Participation/ or decision support techniques/ or (decision mak* or joint working or decision aid* or decision box*).ti,ab,kw. or (shar* adj3 decision*).ti,ab,kw. or (patient* adj (decision* or orientation* or involvement*)).ti,ab,kw. or (shar* adj (information* or care)).ti,ab,kw. or (decision support adj (technique* or system* or method* or intervention*)).ti,ab,kw. |
| 2 | aged/ or "aged, 80 and over"/ or frail older patients/ or exp Aging/ or Geriatrics/ or (elder* or older people* or older person* or older adult* or older patient* or old people* or old person* or old patient* or old adult* or frail* or geriatric* or ag?ing or senior*).ti,ab,kw. |
| 3 | Comorbidity/ or Chronic Disease/ or (comorbidit* or multimorbidit* or co-morbidit* or multi-morbidit*).ti,ab,kw. or (multiple adj (condition* or diseas*)).ti,ab,kw. or frail elder*.ti,ab,kw. or (complex* adj3 (care or healthcare)).ti,ab,kw. or (chronic*.af. and (disease* or condition* or ill*).ti,ab,kw.) |
| 4 | communication barriers/ or (barrier* or facilitator* or obstacle* or hurdle* or hindrance* or limitat* or enabl* or implement* or perceiv* or involv* or challeng* or opportunit* or expectation* or interference*).ti,ab,kw. or (lack* adj3 (knowledge or time)).ti,ab,kw. |
| 5 | 1 and 2 and 3 and 4 |
| 6 | comment/ or editorial/ or letter/ or news/ or (editorial* or comment* or letter* or systematic review*).ti,ab,kw. |
| 7 | 5 not 6 |
| 8 | limit 7 to ((dutch or english or german) and yr="1980 -Current") |

List of key articles used by the development of the search strategy:

1. Frosch DL, Singer KJ, Timmermans S: Conducting implementation research in community-based primary care: a qualitative study on integrating patient decision support interventions for cancer screening into routine practice. Health Expect 2011, 14(Suppl 1):73-84.
2. Uy V, May SG, Tietbohl C, Frosch DL: Barriers and facilitators to routine distribution of patient decision support interventions: a preliminary study in community-based primary care settings. Health Expect 2012, 1-12, Epub ahead of print
3. Stacey D, Graham I, O’Connor A, Pomey M: Barriers and facilitators influencing call center nurses’ decision support for callers facing values sensitive decisions: a mixed methods study. Worldv Evid-Based Nu 2005, 2:184-195.
4. Holmes-Rovner MV, Valade D, Orlowski C, Draus C, Nabozny-Valerio B, Keiser S: Implementing shared decision-making in routine practice: barriers and opportunities. Health Expect 2000, 3:182-191.
5. Frosch DL, Legare F, Mangione CM: Using decision aids in community based primary care: An evaluation with ethnically diverse patients. Patient Educ Couns 2008, 73(3):490-496
6. Cabana MD, Rand CS, Powe NR, Wu AW, Wilson MH, Abboud F P.-A.C.. et al. Why don’t physicians follow clinical practice guidelines? A framework for improvement. J Am Med Assoc 1999;282:1458–65.
7. Holmes-Rovner M, Valade D, Orlowski C, Draus C, Nabozny-Valerio B, Keiser S. Implementing shared decision-making in routine practice: barriers and opportunities. Health Expect 2000;3:182–91.
8. Edwards A, Elwyn G, Wood F, Atwell C, Prior L, Houston H. Shared decision making and risk communication in practice: a qualitative study of GPs’ experiences. Brit J Gen Pract 2005;55:6–13.
9. Wetzels R, Geest TA, Wensing M, Ferreira PL, Grol R, Baker R. GPs’ views on involvement of older patients: an European qualitative study. Patient Educ Couns 2004;53:183–8.
10. Schulman-Green DJ, Naik AD, Bradley EH, McCorkle R, Bogardus ST. Goal setting as a shared decision-making strategy among clinicians and their older patients. Patient Educ Couns 2006;63:145–51
11. Legare F, O’Connor AM, Graham ID, Saucier D, Cote L, Blais J, et al. Primary health care professionals’ views on barriers and facilitators to the implementation of the Ottawa Decision Support Framework in practice. Patient Educ Couns 2006;63:380–90
12. Aasen EM, Kvangarsnes M, Heggen Kr. Perceptions of patient participation amongst elderly patients with end-stage renal disease in a dialysis unit. Scand J Caring Sci 2012;26:61–9.
13. Bastiaens H, Van Royen P, Pavlic DR, Raposo V, Baker R. Older people’s preferences for involvement in their own care: a qualitative study in primary health care in 11 European countries. Patient Educ Couns 2007;68:33–42.
14. Belcher VN, Fried TR, Agostini JV, Tinetti ME. Views of older adults on patient participation in medication-related decision making. J Gen Intern Med 2006;21:298–303.
15. Ekdahl AW, Andersson L, Friedrichsen M. ‘They do what they think is the best for me.’ Frail elderly patients’ preferences for participation in their care during hospitalization. Patient Educ Couns 2010;80:233–40. [46] Ekdahl AW, Andersson L, Wire´hn AB, Friedrichsen M. Are elderly people with co-morbidities involved adequately in medical decision making when hospitalised? A cross-sectional survey. BMC Geriatr 2011;11.
16. Ruan J, Lambert V. Differences in perceived communication barriers among nurses and elderly patients in China. Nurs Health Sci 2008;10:110–6.
17. Fried, T. R., Tinetti, M. E., & Iannone, L. (2011). Primary care clinicians' experiences with treatment decision making for older persons with multiple conditions. *Archives of internal medicine*, *171*(1), 75-80.
18. Schuling, J., Gebben, H., Veehof, L. J. G., & Haaijer-Ruskamp, F. M. (2012). Deprescribing medication in very elderly patients with multimorbidity: the view of Dutch GPs. A qualitative study. *BMC family practice*, *13*(1),
19. Reuben, D. B., & Tinetti, M. E. (2012). Goal-oriented patient care—an alternative health outcomes paradigm. *New England Journal of Medicine*,*366*(9), 777-779. –
20. Ekdahl AW, Andersson L, Wire´hn AB, Friedrichsen M. Are elderly people with co-morbidities involved adequately in medical decision making when hospitalised? A cross-sectional survey. BMC Geriatr 2011;11.
