# Supplementary material for: Barriers and facilitators for shared decision making in older patients with multiple chronic conditions: a systematic review
Source: BMC Geriatr. 2021 Feb 6;21:112. doi: 10.1186/s12877-021-02050-y (PMC7866443; doi:10.1186/s12877-021-02050-y)
Supplement: Supplementary file 2 — Additional file 2: Supplementary Table S2. Taxonomy of barriers and facilitators [file 12877_2021_2050_MOESM2_ESM.docx]

# Supplementary Table S2: Original taxonomy of barriers and facilitators

|  |  | **Barriers** | **Facilitators** |  |
| --- | --- | --- | --- | --- |
| **Predisposing factors** | Patient characteristics | Being in poor health Cognitive/physical impairments Age Ethnicity Lower level of education Poor articulation Difference in personal characteristics Health condition - stigma/discrimination  Long term patient | Being in good health Prior exposure to illness/decision making point Long term patient |  |
|  | Decision characteristics | Timing along the illness trajectory Minor decision Major decision Embarrassing or sensitive topics Shock of receiving diagnosis | Timing along the illness trajectory  Minor decision Major decision Time to come to terms with diagnosis |  |
| **Interactional context factors** | Power (im)balance in the patient -clinician relationship | Presumptions about the patient role  Expectation of the clinician making the decisions  Desire to act like 'good' patient driven by fear of consequences  Perceived acceptability of asking the clinician questions  Belief that health professionals do not want patients involved  Health professionals reinforces passivity by rewarding the behavior  Not having explicit 'permission' to participate in SDM  Patients undervalue their expertise relative to health professionals  'Doctor knows best' and patients have 'inferior' knowledge  Patients are not capable of understanding medical/technical information | Presumptions about the patient role  Perceived acceptability of asking the clinician questions  Having explicit 'permission' to participate in SDM  Patients undervalue their expertise relative to health professionals  Recognizing there a two experts in medical encounter |  |
|  | | Interpersonal characteristics of the clinician | Authoritarian HCP's  Clinician does not listen to patients concerns Health professionals with poor interpersonal skills  Poor relationship with clinician Lack of individualized approach and not asked about preferences Clinician does not address patient directly | Equal relationship  Clinician listens to patients concerns Health professionals with positive interpersonal skills Good relationship with clinician Individualized approach where clinician seeks patient's preferences |
|  | | Trust | Trust in clinician  Lack of trust in clinician | Trust in clinician Lack of trust in clinician |

|  |  | | **Barriers** | **Facilitators** |
| --- | --- | --- | --- | --- |
| **Preparation for a SDM encounter** | | Perceived need for preparation | Patient is not entitled to a choice  Patient is not explicitly offered a choice/or it is presented in a biased way  'Doing nothing' is not an option Patient does not *want* to or *need* to participate in SDM Not knowing what to expect from the SDM consultation | Accepting responsibility to be involved in decision-making |
|  | | Expectation of outcome of being involved in SDM | Not wanting responsibility for wrong decision Fear of accepting reality of diagnosis | Recognizing equipoise and uncertainty |
| **Preparation for the SDM process** | | Providing information about options | Insufficient information about condition, options and outcomes  Clinician does not explain the options and outcomes | Sufficient information about condition, options and outcomes  Clinician explains the options and outcomes |
|  | | Terminology used by HCP's | Clinician uses medical terminology | Clinician uses simple terminology |
|  | | Decision support | Lack of written decision support  Purpose of decision support tool is unclear | Written decision support Decision support from others (e.g. family, other profs) |
| **Social context** | | View of colleagues | Disagreement between colleagues Degree of contact between colleagues Hierarchical structure of professionals |  |
|  | | Culture of network | Social norms and values | Social norms and values |
|  | | Collaboration | Degree of cooperation and response between colleagues | Degree of cooperation and response between colleagues |
|  | | Leadership and social learning | Lack of support from management (incentive, feedback, role models) | Support from management (incentive, feedback, role models) |
| **Organizational context** | | Organizational characteristics | Complexity of the organization |  |
|  | | Capacities | No arrangements for continuous learning | Continuous learning opportunities |
|  | | Organizational constraints | Lack of support services Lack of resources: time Lack of resources: staff | Support services Resources: time Resources: staff |
| **Economic and political context** | | Policy |  | Attractiveness of innovation by means of financial arrangements |
