# Supplementary material for: Barriers and facilitators for shared decision making in older patients with multiple chronic conditions: a systematic review
Source: BMC Geriatr. 2021 Feb 6;21:112. doi: 10.1186/s12877-021-02050-y (PMC7866443; doi:10.1186/s12877-021-02050-y)
Supplement: Supplementary file 3 — Additional file 3: Supplementary Table S3. Quality assessment of included studies [file 12877_2021_2050_MOESM3_ESM.docx]

# Supplementary Table S3: Quality assessment of included studies (according to the Standard Quality Assessment Criteria ([Kmet, Lee, & Cook, 2004](#_ENREF_1))

| Qualitative studies (part 1) |  |  |  |  |  |  |  |  |  |
| --- | --- | --- | --- | --- | --- | --- | --- | --- | --- |
|  |  |  |  |  |  |  |  |  |  |
| Study identification | [(Belcher et al., 2006)](file:///G:\20190214%20review\stap%207%20nieuwe%20versies%20review\quality%20assessment%20new%20totaL.xlsx#RANGE!_ENREF_1) | [(Bragstad et al., 2014)](file:///G:\20190214%20review\stap%207%20nieuwe%20versies%20review\quality%20assessment%20new%20totaL.xlsx#RANGE!_ENREF_2) | [(Dyrstad et al., 2015)](file:///G:\20190214%20review\stap%207%20nieuwe%20versies%20review\quality%20assessment%20new%20totaL.xlsx#RANGE!_ENREF_9) | [(Ekdahl et al., 2012)](file:///G:\20190214%20review\stap%207%20nieuwe%20versies%20review\quality%20assessment%20new%20totaL.xlsx#RANGE!_ENREF_12) | [(Ekdahl et al., 2011)](file:///G:\20190214%20review\stap%207%20nieuwe%20versies%20review\quality%20assessment%20new%20totaL.xlsx#RANGE!_ENREF_11) | [(Ekdahl et al., 2010)](file:///G:\20190214%20review\stap%207%20nieuwe%20versies%20review\quality%20assessment%20new%20totaL.xlsx#RANGE!_ENREF_10) | [(Fried et al., 2011)](file:///G:\20190214%20review\stap%207%20nieuwe%20versies%20review\quality%20assessment%20new%20totaL.xlsx#RANGE!_ENREF_17) | [(Gauthier, 2005)](file:///G:\20190214%20review\stap%207%20nieuwe%20versies%20review\quality%20assessment%20new%20totaL.xlsx#RANGE!_ENREF_19) | [(Lindhardt et al., 2008)](file:///G:\20190214%20review\stap%207%20nieuwe%20versies%20review\quality%20assessment%20new%20totaL.xlsx#RANGE!_ENREF_31) |
|  |  |  |  |  |  |  |  |  |  |
| Question / objective sufficiently described | 2 | 2 | 2 | 2 | 2 | 2 | 2 | 2 | 2 |
| Study design evident and appropriate? | 2 | 2 | 2 | 2 | 2 | 2 | 2 | 2 | 2 |
| Context for the study clear? | 2 | 1 | 2 | 2 | 2 | 2 | 2 | 1 | 2 |
| Connection to a theoretical framework / wider body of knowledge? | 2 | 2 | 2 | 2 | 2 | 2 | 2 | 2 | 2 |
| Sampling strategy described, relevant and justified? | 1 | 2 | 1 | 1 | 2 | 1 | 2 | 1 | 2 |
| Data collection methods clearly described and systematic? | 2 | 2 | 2 | 2 | 2 | 2 | 2 | 1 | 2 |
| Data analysis clearly described and systematic? | 2 | 2 | 2 | 2 | 2 | 2 | 2 | 2 | 2 |
| Use of verification procedure(s) to establish credibility? | 2 | 2 | 2 | 2 | 0 | 2 | 2 | 0 | 2 |
| Conclusions supported by the results? | 2 | 2 | 2 | 2 | 2 | 2 | 2 | 2 | 2 |
| Reflexivity of the account? | 0 | 0 | 2 | 1 | 0 | 2 | 0 | 0 | 2 |
| Total score /possible maximum score | 17/20 | 17/20 | 19/20 | 18/20 | 16/20 | 19/20 | 18/20 | 13/20 | 20/20 |
| Summary score (0-1) | 0.85 | 0.85 | 0.95 | 0.90 | 0.80 | 0.95 | 0.90 | 0.65 | 1.00 |

| Qualitative studies (part 2) |  |  |  |  |  |  |  |  |  |
| --- | --- | --- | --- | --- | --- | --- | --- | --- | --- |
|  |  |  |  |  |  |  |  |  |  |
| Study identification | [(Schuling et al., 2012)](file:///G:\20190214%20review\stap%207%20nieuwe%20versies%20review\quality%20assessment%20new%20totaL.xlsx#RANGE!_ENREF_37) | [(Funk, 2004)](file:///G:\20190214%20review\stap%207%20nieuwe%20versies%20review\quality%20assessment%20new%20totaL.xlsx#RANGE!_ENREF_18) | (Blaum et al, 2018) | (Puts et al, 2017) | (Doekhie et al. 2018) | (Ferris et al. 2018) | (Riffin et al. 2018) | (Peacock et al, 2017) | (Petrillo et al. 2018) |
|  |  |  |  |  |  |  |  |  |  |
| Question / objective sufficiently described | 2 | 2 | 2 | 2 | 2 | 2 | 2 | 2 | 2 |
| Study design evident and appropriate? | 2 | 2 | 1 | 2 | 2 | 2 | 2 | 2 | 2 |
| Context for the study clear? | 1 | 2 | 2 | 2 | 2 | 2 | 2 | 2 | 2 |
| Connection to a theoretical framework / wider body of knowledge? | 2 | 2 | 2 | 1 | 2 | 2 | 1 | 2 | 2 |
| Sampling strategy described, relevant and justified? | 2 | 2 | 2 | 2 | 2 | 2 | 2 | 2 | 2 |
| Data collection methods clearly described and systematic? | 2 | 2 | 0 | 2 | 2 | 2 | 2 | 2 | 2 |
| Data analysis clearly described and systematic? | 2 | 2 | 0 | 2 | 2 | 2 | 2 | 2 | 2 |
| Use of verification procedure(s) to establish credibility? | 2 | 0 | 0 | 2 | 1 | 2 | 1 | 2 | 2 |
| Conclusions supported by the results? | 2 | 2 | 2 | 2 | 2 | 2 | 2 | 2 | 2 |
| Reflexivity of the account? | 0 | 0 | 0 | 0 | 2 | 0 | 0 | 0 | 0 |
| Total score /possible maximum score | 17/20 | 16/20 | 11/20 | 17/20 | 19/20 | 18/20 | 16/20 | 18/20 | 18/20 |
| Summary score (0-1) | 0.85 | 0.80 | 0.55 | .85 | 0.95 | 0.90 | 0.80 | 0.90 | 0.90 |

| Quantitative studies |  |  |  |  |
| --- | --- | --- | --- | --- |
|  |  |  |  |  |
| Study identification | [(Menne & Whitlatch, 2007)](file:///G:\20190214%20review\stap%207%20nieuwe%20versies%20review\quality%20assessment%20new%20totaL.xlsx#RANGE!_ENREF_33) | [(Milte et al., 2015)](file:///G:\20190214%20review\stap%207%20nieuwe%20versies%20review\quality%20assessment%20new%20totaL.xlsx#RANGE!_ENREF_34) | [(Naik et al., 2011)](file:///G:\20190214%20review\stap%207%20nieuwe%20versies%20review\quality%20assessment%20new%20totaL.xlsx#RANGE!_ENREF_36) | (Chi et al. 2017) |
|  |  |  |  |  |
| Question/ objective sufficiently described? | 2 | 2 | 2 | 2 |
| Study design evident and appropriate? | 2 | 2 | 1 | 2 |
| Method of subject/comparison group selection or source of information/input variables described and appropriate? | 2 | 2 | 1 | 2 |
| Subject (and comparison group, if applicable) characteristics sufficiently described? | 2 | 2 | 2 | 2 |
| If interventional and random allocation was possible, was it described? | n/a | 2 | n/a | n/a |
| If interventional and blinding of investigators was possible, was it reported? | n/a | 2 | n/a | n/a |
| If interventional and blinding of subjects was possible, was it reported? | n/a | n/a | n/a | n/a |
| Outcome and (if applicable) exposure measure(s) well defined and robust to measurement / misclassification bias? Means of assessment reported? | 2 | 2 | 2 | 2 |
| Sample size appropriate? | 2 | 2 | 2 | 2 |
| Analytic methods described/justifi ed and appropriate? | 2 | 2 | 2 | 2 |
| Some estimate of variance is reported for the main results? | 2 | 2 | 2 | 0 |
| Controlled for confounding? | 0 | 0 | 0 | 0 |
| Results reported in sufficient detail? | 2 | 1 | 1 | 2 |
| Conclusions supported by the results? | 2 | 2 | 2 | 2 |
|  |  |  |  |  |
| Total score /possible maximum score | 20/22 | 23/26 | 17/22 | 18/22 |
| Summary score (0-1) | 0.91 | 0.88 | 0.77 | 0.80 |

| Mixed Method Studies |  |  |  |
| --- | --- | --- | --- |
|  |  |  |  |
| *Qualitative part* |  |  |  |
|  |  |  |  |
| Study identification | (Legare et al., 2013)* | (Kiselev et al. 2017) | (Rose et al. 2018) |
|  |  |  |  |
| Question / objective sufficiently described | 2 | 2 | 2 |
| Study design evident and appropriate? | 2 | 2 | 2 |
| Context for the study clear? | 2 | 2 | 2 |
| Connection to a theoretical framework / wider body of knowledge? | 2 | 2 | 1 |
| Sampling strategy described, relevant and justified? | 2 | 2 | 2 |
| Data collection methods clearly described and systematic? | 2 | 2 | 2 |
| Data analysis clearly described and systematic? | 2 | 2 | 2 |
| Use of verification procedure(s) to establish credibility? | 2 | 2 | 1 |
| Conclusions supported by the results? | 2 | 2 | 2 |
| Reflexivity of the account? | 2 | 0 | 0 |
| Total score /possible maximum score | 20/20 | 18/20 | 16/20 |
| Summary score (0-1) | 1.00 | 0.90 | 0.80 |
|  |  |  |  |
| *Quantitative part* |  |  |  |
|  |  |  |  |
|  |  |  |  |
| Question/ objective sufficiently described? | 2 | 2 | 2 |
| Study design evident and appropriate? | 2 | 2 | 2 |
| Method of subject/comparison group selection or source of information/input variables described and appropriate? | 2 | 2 | 2 |
| Subject (and comparison group, if applicable) characteristics sufficiently described? | 2 | 2 | 1 |
| If interventional and random allocation was possible, was it described? | n/a | n/a | n/a |
| If interventional and blinding of investigators was possible, was it reported? | n/a | n/a | n/a |
| If interventional and blinding of subjects was possible, was it reported? | n/a | n/a | n/a |
| Outcome and (if applicable) exposure measure(s) well defined and robust to measurement / misclassification bias? Means of assessment reported? | 2 | 2 | 2 |
| Sample size appropriate? | 2 | 2 | 2 |
| Analytic methods described/justified and appropriate? | 2 | 2 | 2 |
| Some estimate of variance is reported for the main results? | 2 | 2 | 2 |
| Controlled for confounding? | 0 | 0 | 0 |
| Results reported in sufficient detail? | 2 | 2 | 2 |
| Conclusions supported by the results? | 2 | 2 | 2 |
| Total score /possible maximum score | 20/22 | 20/22 | 19/22 |
| Summary score (0-1) | 0.90 | 0.90 | 0.86 |
| Summary score mixed methods | 0,95 | 0.90 | 0.83 |
|  |  |  |  |
